# The Influence of Increasing Levels of Provider-Patient Discussion on Quit Behavior: An Instrumental Variable Analysis of a National Survey

**DOI:** 10.3390/ijerph18094593

**Published:** 2021-04-26

**Authors:** Bian Liu, Serena Zhan, Karen M. Wilson, Madhu Mazumdar, Lihua Li

**Affiliations:** 1 Department of Population Health Science and Policy, Icahn School of Medicine at Mount Sinai, New York, NY 10029-6574, USA; Serena.Zhan@mountsinai.org (S.Z.); madhu.mazumdar@mountsinai.org (M.M.); Lihua.Li@mountsinai.org (L.L.); 2 Institute for Translational Epidemiology, Icahn School of Medicine at Mount Sinai, New York, NY 10029-6574, USA; 3 Tisch Cancer Institute, New York, NY 10029-6574, USA; 4 Institute for Healthcare Delivery Science, Icahn School of Medicine at Mount Sinai, New York, NY 10029-6574, USA; 5 Department of Pediatrics, Icahn School of Medicine at Mount Sinai, New York, NY 10029-6574, USA; karen.wilson@mssm.edu

**Keywords:** provider–patient communication, intent to quit, attempt to quit, instrumental variable, smoking cessation

## Abstract

Objective: We aimed to examine the influence of increasing levels of discussion (both asked and advised, either asked or advised but not both, and neither asked nor advised) on quit behavior. Methods: We included 4133 adult current smokers from the 2015 National Health Interview Survey. The primary outcomes were quit intent and quit attempt, and the secondary outcomes were methods used for quitting. We used an instrumental variable analysis, as well as propensity score weighted and multivariable logistic regressions. Results: Compared to no discussion, having both or only one discussion, respectively, increased quit intent (OR = 1.65, 95% CI = 1.63–1.66 and OR = 1.02, 95% CI = 0.99–1.05), quit attempt (OR = 1.76, 95% CI = 1.75–1.77 and OR = 1.60, 95% CI = 1.57–1.63). Among those who attempted to quit (n = 1536), having both or only one discussion increased the use of pharmacologic (OR = 1.99, 95% CI = 1.97–2.02 and OR = 1.56, 95% CI = 1.49–1.63) or behavioral (OR = 2.01, 95% CI = 1.94–2.08 and OR = 2.91, 95% CI = 2.74–3.08) quit methods. Conclusions: Increasing levels of provider–patient discussion encourages quit behavior, and should be an integral part of reducing the health and economic burden of smoking. Strategies that promote the adherence and compliance of providers to communicate with patients may help increase the success of smoking cessation.

## 1. Introduction

While the prevalence of cigarette smoking has been falling and reached an all-time low of 14% among the U.S. adult population, smoking remains the leading preventable behavior for many major diseases including cancer and cardiovascular diseases [1]. To encourage behavior change, the U.S. Public Health Service’s Clinical Practice Guideline recommends healthcare professionals to use the 5A’s approach to help smokers quit: (1) ask about tobacco use at every visit; (2) advise all tobacco users to quit; (3) assess readiness to quit; (4) assist tobacco users with a quit plan; and (5) arrange follow-up visits [2,3,4].

Despite this five-step guideline, and studies showing positive effects of a provider’s discussion in helping smokers quit, the uptake of provider–patient discussion remains suboptimal [1,5]. Based on nationally representative samples from the National Health Interview Survey (NHIS), the prevalence of provider–patient discussion about smoking (the first A: ask) ranged from 51.3% in 2011 to 55.4% in 2015 [6,7]. The prevalence of patient–provider discussion on quitting (the second A: advise) ranged from 53.3% in 2000, and 58.9% in 2005, to 50.7% in 2010, and was 57.2% in 2015 [8,9]. Multiple factors influence the uptake of provider–patient discussion, including patient’s sex, age, race/ethnicity, education, health insurance, and health conditions [6,10]. For example, Hispanics were found to be less likely to receive advice to quit than non-Hispanic white people [8,11,12,13]. Older adults with smoking-related cancer were more likely to be advised to quit smoking by a healthcare provider than those without chronic diseases [14].

The levels of provider–patient discussion of the recommended 5A’s also vary in different settings, which in turn can affect quit behavior. In the National Adult Tobacco Survey (NATS), where all 5A’s were available, the proportion of ask, advise, assess, assist, and arrange was 88.3%, 66.4%, 43.4%, 38.6%, and 6.3%, respectively [15]. Corroborating results were reported in other studies showing that the compliance of provider–patient discussion decreased with subsequent 5A’s steps [5,16,17,18]. However, investigations on the combined usage of individual levels of the 5A’s using NATS data were limited by the small sample sizes available, where the proportion of patients who received any one of the 5A’s was 33.6%, any two was 18.3%, any three was 13.9%, any four was 28.1%, and all five was only 6.1% [15]. Existing studies that used NHIS data, where only the first two A’s of the five-step algorithm were available, focused on either the ask or advice aspect alone [6,13,14,19,20,21,22,23]. No study based on NHIS has examined the impact of increasing levels of provider–patient discussion (ask and advice, advice alone or ask alone, as compared to no discussion) on quit behavior. 

In the current study, we aimed to investigate whether there was an increasing level of the positive influence of provider–patient discussion with the first 2A’s on quit behavior based on NHIS data. We also extended the existing studies with a rigorous statistical approach by using an instrumental variable (IV) analysis to reduce estimate bias due to unobserved confounders. To complement the IV analysis, we conducted multivariable logistic regressions and propensity score-based weighting regressions. Results from the study may help improve our understanding of the influence of provider–patient discussion and inform policy and practice on promoting smoking cessation. 

## 2. Methods

### 2.1. Data Sources and Study Population 

We used publicly available datasets from the 2015 NHIS, an ongoing cross-sectional survey. Each year, NHIS interviews a representative sample of the civilian non-institutionalized population in the U.S. through a multistage probability design [24]. We used both the core survey components and the latest available Tobacco Section in the Cancer Control Supplement, which was from 2015, to obtain comprehensive information about current smokers’ smoking and quit behavior, as well as their interactions with healthcare providers. 

We included 4133 participants in the analysis following detailed selection steps outlined in the Supplementary Figure S1. Briefly, we first identified 5415 adult (age ≥ 18 years) current smokers in the 2015 NHIS data. Current smokers were defined as individuals who smoked at least 100 cigarettes in their lifetime and currently smoke cigarettes daily or on some days. We further restricted to those who had seen a doctor or other health professional in the past 12 months. We also excluded participants with missing values and those who responded “don’t know” or “refused” for the main outcomes, exposure, and covariates.

### 2.2. Outcome Variables

We focused on two binary (yes/no) primary outcomes: intent to quit (would like to completely quit smoking cigarettes) and attempt to quit (stopped smoking for more than one day because of trying to quit smoking during the past 12 months). Among those who attempted to quit, we further grouped the quit methods into pharmacological and behavioral methods, which were secondary outcomes. The pharmacological cessation method included self-reported use of any of the following: Chantix or Varenicline, Zyban, Bupropion, or Wellbutrin, nicotine patch, nicotine gum or lozenge, or nicotine containing nasal spray or inhaler. The behavioral cessation method included self-reported use of any the following: telephone help/quit line, one-on-one counseling, stop smoking clinic, class, or support group. 

### 2.3. Exposure Variables

From the same cross-sectional survey, we defined the exposure variable as a 3-level provider–patient discussion being both asked and advised, either asked or advised but not both, or neither asked nor advised, based on participants’ responses to the following two questions: “DURING THE PAST 12 MONTHS, has a doctor or other health professional talked to you about your smoking?”, and “In the PAST 12 MONTHS, has a medical doctor, dentist, or other health professional ADVISED you to quit smoking, or to quit using other kinds of tobacco?”. We grouped only asked and only advised into the same category, assuming they had equivalent impact on quit intent and attempt.

### 2.4. Covariates

We included the following commonly adjusted socio-demographic factors: age, sex, race/ethnicity, marital status, and region. We also included education, employment status, health insurance type, and the ratio of family income to the applicable federal poverty thresholds. We then included health status variables that are indicative of the following smoking-related comorbidities: lung disease, cardiovascular diseases, and cancer [25]. Similar to previous studies [14,26], we included an indicator for serious psychological distress using the Kessler Scale. In addition, we included two measures of smoking exposure: years smoked, and numbers of cigarettes smoked daily. The former was calculated by subtracting age at smoking initiation from age at interview, while the latter was estimated based on answers to the following question: “On the average, when you smoked during the past 30 days, about how many cigarettes did you smoke a day?”. In total, we included 16 covariates for the analysis.

### 2.5. Instrumental Variable

Cross-sectional studies are prone to biases and confounders. While adjusting for potential confounders in multivariable regression models can ameliorate the problem, unobserved confounders may still exist. One approach to overcome this limitation is the use of an instrumental variable (IV). Different from a confounding variable, which is associated with both the outcome and the exposure, IV is a factor that is associated with the exposure but not with the outcome, and the effect of IV on the outcome is through the exposure. Due to the infeasibility of randomly assigning the subjects to groups as in randomized control trials (RCTs), an IV analysis is often applied in observational studies to yield an unbiased estimate of the effect of exposure on the outcome [27,28]. 

**Figure 1:**
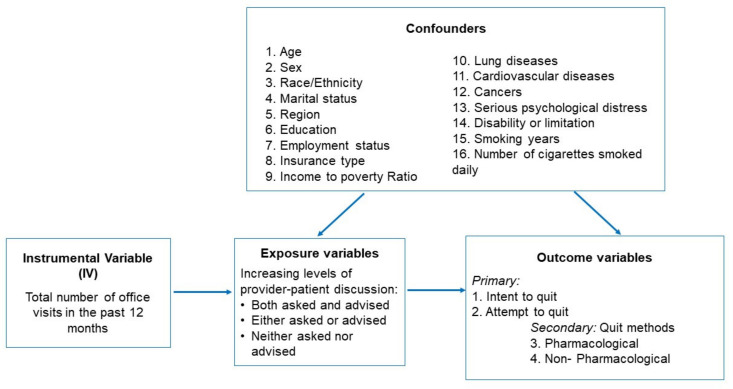
A graphic representation of the instrumental variable (IV) analysis.

We considered the number of office visits in the past 12 months as the IV (Figure 1), with the rationale that the more office visits a patient has, the higher chance of him or her having a provider–patient discussion about smoking/quitting, which in turn can lead to a higher likelihood of smoking cessation. 

### 2.6. Statistical Analysis

All analyses were performed using the SAS Enterprise Guide (version 7.1, SAS Institute Inc., Cary, NC, USA) and R version 3.6.2, during January to May 2020. Odds ratio (ORs) and 95% confidence interval (CI) were reported for the exposure–outcome association. All *p*-values were two-sided and a value less than 0.05 was considered statistically significant. Unless otherwise specified, all the analyses took into account the complex NHIS survey design to provide weighted population-level estimates. 

In the descriptive analysis, we compared the respondents’ characteristics by the 3-level patient–provider discussion on smoking and quitting, using the Rao–Scott Chi-squared test for categorical variables and the F test for continuous variables [29]. Supplementary Table S1 lists the unweighted sample characteristics.

#### 2.6.1. Instrumental Variable (IV) Analysis

We applied a two-stage residual inclusion (2SRI) method, an established method for producing consistent estimators for nonlinear parametric models [30,31]. In the first stage, the provider–patient discussion was modeled as a function of the IV and the aforementioned covariates using a multinomial logistic regression. In the second stage, the individual dichotomized outcome variable was predicted as a function of the exposure variable, all covariates, and the standardized residuals obtained from the first stage using a binomial logistic regression. The standard errors of two-stage estimators were calculated by implementing a bootstrap method with 500 repetitions [30,31].

We also performed the first-stage F test and falsification test to check the validity of two assumptions for IV analysis. First, an IV must be correlated with the exposure variable; second, the IV must not be correlated with the outcome variable or any other unmeasured confounders such that the effect of the IV on the outcome is only through the exposure variable [32]. The first assumption is often tested using the first-stage F test, where an IV is not considered a weak instrument if the value of the F statistic is greater than 10 [33]. Since the second assumption cannot be directly tested through a confirmatory test, a falsification test is performed as an alternative to show empirically a lack of violation [34]. A *p*-value of the coefficient of IV < 0.05 indicates that the IV may have direct effect on the outcome.

#### 2.6.2. Sensitivity Analysis

We performed two sensitivity analyses using a propensity score weighting (PSW) model and a multivariable logistic model to ensure the robustness of the estimated effects from the IV analysis. The PSW approach is often used for an unbiased estimate when a RCT is not feasible [35,36]. We first derived a propensity score using a multinomial logistic regression, where the propensity score was the probability of having a provider–patient discussion conditioned on the covariates. Then, we modeled the association between the provider–patient discussion and quit behavior outcomes using a survey logistic regression, where the weight variable was the product of survey weight and the propensity score weight [37,38].

## 3. Results

### 3.1. Sample Characteristics

Out of 4133 adult current smokers who visited a doctor or a healthcare provider during the past 12 months, 735 (weighted prevalence: 17.61%) reported either being asked by the provider about smoking or advised to quit smoking, and 2219 (weighted prevalence: 54.64%) reported being both asked and advised (Table 1). Compared to those who had neither discussion, respondents who had both or only one level discussion were more likely to be older (mean age: 47.69 years and 44.23 years vs. 40.88 years, *p*-value < 0.001), female (51.26% and 55.21% vs. 43.14%, *p*-value < 0.001), married (56.16% and 50.36% vs. 48.79%, *p*-value = 0.007), and residing in the Northeast of the U.S. (19.07% and 13.67% vs. 12.23%, *p*-value < 0.001). They were less likely to be Hispanic (6.22% and 8.88% vs. 14.61%, *p*-value < 0.001), or without health insurance (10.23% and 13.28% vs. 21.70%, *p*-value < 0.001), and less likely to be employed (54.53% and 56.79% vs. 66.43%, *p*-value < 0.001). They were also more likely to have smoking-related comorbidities (*p*-value < 0.001), serious psychological distress (13.43% and 9.69% vs. 6.61%, *p*-value < 0.001), and disability or activity limitations (31.38% and 23.75% vs. 12.90%, *p*-value < 0.001). Moreover, they had a higher level of smoking exposure (*p*-value < 0.001).

### 3.2. Associations between Provider-Patient Discussion and Quit Behavior

The population-weighted proportions of having quit intent were 65%, 66%, and 74% among those who were neither asked nor advised, either asked only or advised only, and both asked and advised, respectively; of having quit attempts were 46%, 47%, and 54%, respectively; for using pharmacological quit methods were 18%, 31%, and 40%, respectively; and, for using non-pharmacological quit methods were 4%, 11%, and 10%, respectively.

Compared to receiving neither asked nor advised discussion, being both asked and advised was associated with greater odds for quit intent (OR 1.65, 95% CI (1.63−1.66)) and quit attempt (OR 1.76, 95% CI (1.75–1.77)); while being either asked or advised was only statistically significantly associated with quit attempt (OR 1.60, 95% CI (1.57–1.63)), but not with quit intent (OR 1.02, 95% CI (0.99–1.05)) (Table 2).

Among those who tried to quit, having both or only one level discussion was significantly associated with the use of pharmacological or non-pharmacological quit methods, as compared to having no discussion (Table 2). The association in pharmacological quit methods was slightly higher for having both rather than only one discussion (OR = 1.99, 95% CI (1.97–2.02) vs. 1.56, 95% CI (1.49–1.63)). The opposite trend was found for non-pharmacological quit methods, where the association was lower for having both rather than one discussion (OR 2.01, 95 % CI (1.94–2.08) vs. OR 2.91, 95% CI (2.74−3.08)).

Both the F test and the falsification test for assumption checking indicated that the number of doctor office visits was a reasonable instrumental variable (Supplementary Table S2).

Results from the PSW and multivariable logistic regression were consistent with those observed in the main IV analysis (Table 3). Supplementary Table S3 shows the comparison of sample characteristics before and after PSW.

## 4. Discussion

The use of 5A’s has long been recognized as an evidence-based guideline for healthcare providers to promote smoking cessation among current smokers; however, its effectiveness varies [10,39]. Using a nationally representative sample of non-institutionalized adults, we found that current smokers who reported being both asked and advised by a healthcare provider had significantly increased odds (65%) of quit intent, compared to those who reported receiving neither discussion, while the increase was not significant among smokers who reported being only asked or only advised. Having both discussions also had a higher point estimate for quit attempt than having one discussion. For those who tried to quit, the odds of trying to quit with the assistance of pharmacological or non-pharmacological methods also increased among those with a provider–patient discussion, which is consistent with the known effectiveness of these two cessation methods [40,41]. In addition, we found a higher association in choosing pharmacological quit methods among those who received both rather than single discussion. It was also encouraging to find that about 50% of current smokers had tried to quit.

The finding of a growing influence between increasing levels of provider–patient discussion on quit behavior is new, as no NHIS studies have examined such a relationship. This finding suggests that delivery of both ask and advise may encourage more quit attempts among current smokers than delivery of only one of these 2A’s, and even having one of the 2A’s is better than no discussion at all. Our result supports the notion that each provider–patient discussion presents an opportunity to promote smoking cessation, and combined discussions may achieve a greater effect. This study provides concrete evidence that improving provision of even just one of the first 2A’s of the physician–patient discussion guideline can increase the probability of smoking cessation.

Regardless of the use of both of the first 2A’s or just one, having any level of provider–patient discussion was statistically significantly associated with increased quit attempts and greater use of either pharmacological or behavioral quit methods. Our results agreed with previous findings based on NHIS data that did not differentiate between the degrees of discussion [6,13,14,19,20,21,22,23]. Our result is also consistent with studies based on NATS data where all 5 A’s were surveyed, which showed that smokers who received any three or four components of the 5A’s were associated with greater use of cessation treatments [15]. Different from the previous studies, we detected some discrepancies in the method chosen by the levels of provider–patient discussion. Current smokers who received both 2A’s tended to choose pharmacological quit methods, while those who received either of the 2A’s tended to choose non-pharmacological quit methods. It is possible that those who received 2A’s might have received other A’s which were not asked in NHIS, though another study indicated a progressive decline in reports of receiving the 5A’s from first to last [15]. Given the evidence that using cessation medication approved by the FDA alone or in conjunction with behavioral counseling increases the chance of cessation [40,41], our results highlight the importance of having a comprehensive provider–patient discussion in helping smokers quit.

This study had several methodological strengths. Our IV analysis reduced the bias in estimating the association between provider–patient discussion and quit behavior compared to the estimates from commonly used multivariable logistic regressions. Biased estimates resulting from unobserved confounders are a major concern in observational studies [42], and their impact can be mitigated through an IV analysis. Candidates for IV in the context of this and similar studies can be distance-, time-, or preference-based [10,43]. Previously, Bao et al. used physicians’ advice about diet and physical activity as the IV to address the selection bias of a provider in giving smoking cessation advice [10]. Different from that study, we used the number of office visits as the IV, with the rationale that patients with more visits tend to be asked/advised more, and this patient behavior pattern is not directly correlated with quit behavior. Results from our IV analysis were consistent with that from the PSW regression, another sophisticated method to reduce confounding and unobserved bias [36]. The comparison of three approaches demonstrated the consistency and strong internal validity of our results.

Our study also had a few limitations. First, the responses were based on self-reported data, such as comorbidities, which were not confirmed by medical record. The outcomes of interest were also subject to recall bias [6]. Due to the nature of cross-sectional NHIS data, we were not able to establish temporality or draw causal inference. For instance, the survey lacked information about whether the intent/attempt to quit smoking occurred after being asked/advised by a provider. We also lacked the detailed information about the content and timing of the provider–patient discussion or about the provider’s characteristics, all of which could affect smokers’ quit behavior and choice of cessation methods. We were only able to assess the first 2A’s of the five-step algorithm, as other components (assess, assist, and arrange) were not surveyed in the NHIS. We could not rule out the possibility that some participants who had one or both of the first 2A’s might also have some or all of the other A’s, though the portion of these patients was likely to be small as shown in a national survey where data on all 5A’s were available [15]. Lastly, as the landscape of tobacco use has changed rapidly in recently years, [44] so has the provider–patient communication about smoking. While we used the most up-to-date publicly available data, more recent population-based data on the 5A’s are needed.

## 5. Conclusions

Smoking remains the leading cause of preventable diseases and deaths in the United States. Approximately 34 million adults currently smoke cigarettes, 70% of whom say they want to quit [1]. Our findings shed light on understanding the influence of varying levels in provider–patient discussion on the patient’s smoking cessation behavior. Being asked and/or advised has consistently increased smokers’ quit intent and quit attempt, as well as their use of pharmacological or behavioral cessation methods, compared to no provider–patient discussion. Individuals who currently smoke cigarettes may benefit more from increased levels of being asked and advised by a provider than either asked or advised alone.

Strategies that facilitate the adherence and compliance of providers to communicate with patients, such as continued education/training, embedding the 5A’s algorithm or three-step methods (e.g., ask-advise-refer or ask-advise-connect [45,46,47]) in the electronic health record, and adding incentives for both parties to promote provider–patient discussion, may help increase the success of smoking cessation [18,41,48,49].

## Figures and Tables

**Table 1:** Characteristics of the study sample by the level of provider–patient discussion, 2015 NHIS.

Levels of Discussion	Asked and Advised (*n* = 2219)	Asked or Advised (*n* = 735)	Neither Asked or Advised (*n* = 1179)	
**Categorical Variables**	***N* (weighted%)**	***N* (weighted%)**	***N* (weighted%)**	***p*-value ^a^**
Sex				<0.001
Female	1202 (51.26)	414 (55.2)	563 (43.14)	
Male	1017 (48.74)	321 (44.8)	616 (56.86)	
Race/Ethnicity				<0.001
White Non-Hispanic	1591 (78.09)	490 (71.5)	753 (65.36)	
Black Non-Hispanic	310 (11.29)	135 (15.58)	150 (12.68)	
Hispanic	197 (6.22)	68 (8.88)	172 (14.61)	
Others	121 (4.4)	42 (4.03)	104 (7.34)	
Marital Status ^b^				0.007
Yes	958 (56.16)	287 (50.36)	464 (48.79)	
No	1261 (43.84)	448 (49.64)	715 (51.21)	
Region				<0.001
Northeast	405 (19.07)	95 (13.67)	148 (12.23)	
Midwest	535 (29.11)	173 (24.47)	305 (26.8)	
South	772 (35.05)	287 (41.69)	408 (38.03)	
West	507 (16.77)	180 (20.18)	318 (22.94)	
Education				0.706
Less than High School	538 (24.03)	205 (28.37)	284 (25.3)	
High School Graduate	598 (26.65)	191 (26.69)	300 (26.21)	
Some College	519 (23.29)	170 (22.93)	292 (23.43)	
College or Above	564 (26.03)	169 (22.01)	303 (25.07)	
Employment				<0.001
Yes	1083 (54.53)	397 (56.79)	737 (66.43)	
No	1136 (45.47)	338 (43.21)	442 (33.57)	
Insurance				<0.001
Private	806 (44.17)	288 (42.54)	505 (47.36)	
Medicaid	434 (17.54)	153 (21.62)	215 (17.18)	
Medicare	555 (19.5)	147 (17.61)	134 (8.76)	
Others	210 (8.56)	52 (4.95)	61 (5)	
Uninsured	214 (10.23)	95 (13.28)	264 (21.7)	
Income/poverty ratio				0.860
<1.00	576 (19.42)	191 (22.56)	307 (21.51)	
1.00–1.99	569 (23.92)	194 (23.5)	288 (23.14)	
2.00–3.99	633 (31.27)	206 (29.95)	343 (31.71)	
4.00 and over	441 (25.38)	144 (23.99)	241 (23.65)	
Lung Disease ^c^				<0.001
Yes	1279 (53.09)	312 (38.24)	386 (29.27)	
No	940 (46.91)	423 (61.76)	793 (70.73)	
CVD ^d^				<0.001
Yes	310 (12.07)	65 (7.83)	63 (4.29)	
No	1909 (87.93)	670 (92.17)	1116 (95.72)	
Cancer ^e^				<0.001
Tobacco Related	87 (3.09)	17 (2.13)	20 (0.93)	
Non-tobacco Related	152 (6.3)	43 (4.66)	33 (2.68)	
None	1980 (90.61)	675 (93.21)	1126 (96.39)	
Serious psychological distress ^f^				<0.001
Yes (Kessler score ≥ 13)	272 (13.43)	76 (9.69)	82 (6.61)	
No (Kessler score < 13)	1947 (86.57)	659 (90.31)	1097 (93.39)	
Disability/limitation ^g^				<0.001
Yes	854 (31.38)	196 (23.75)	205 (12.9)	
No	1365 (68.62)	539 (76.25)	974 (87.1)	
**Continuous variables**	**Weighted Mean (SD)**	**Weighted Mean (SD)**	**Weighted Mean (SD)**	***p*-value ^a^**
Age (years)	47.69 (14.74)	44.23 (15.9)	40.88 (15.12)	<0.001
Smoking length ^h^ (year)	29.9 (15.19)	26.06 (16.12)	22.59 (15.37)	<0.001
Number of cigarettes smoked daily	13.37 (9.32)	11.08 (9.25)	9.57 (8.07)	<0.001

**Notes:**^a^ The analysis took into account the NHIS survey design. Comparison of categorical variables by the 3-level provider–patient discussion was conducted using the Rao–Scott Chi-square test in SURVEYFREQ procedure, and using the F-test in SURVEYREG procedure. ^b^ Married included those who are married or living with a partner. ^c^ Lung disease types included COPD, emphysema, and chronic bronchitis. ^d^ Cardiovascular diseases (CVD) included coronary heart disease, angina, stroke hypertension, heart attack, and other heart disease. ^e^ Tobacco-related cancer types included 12 tobacco-associated cancers as defined by the CDC: lip, oral cavity, pharynx, esophagus, stomach, colon and rectum, liver, pancreas, larynx, trachea, lung, bronchus, cervix uteri, kidney and renal pelvis, urinary bladder, and acute myeloid leukemia. ^f^: The Kessler Psychological Distress Scale consists of six questions that ask about feelings of sadness, nervousness, restlessness, worthlessness, hopelessness, and feeling like everything is an effort during the past 30 days. Participants were asked to respond on a Likert Scale ranging between ‘none of the time’ (score = 0) to ‘all of the time’ (score = 4), and a cutoff of 13 was used to dichotomize the status of serious psychological distress. ^g^ Defined as having any functional limitations inclusive of all physical conditions. ^h^ Calculated as the difference between age at interview and age when smoking regularly.

**Table 2:** The association between provider–patient discussion and quit behavior outcomes: quit intent, quit attempt, and quit methods from an instrumental variable analysis.

Outcome Variable	Exposure Variable: Level of Discussion	Outcome Events/Total	Weighted % of Outcome Events	Odds Ratio (95% CI)
Intent to Quit (Yes vs. No)	Both Asked and Advised	1611/2184	74%	1.65 (1.63–1.66) Funding:
	Either Asked or Advised	479/727	66%	1.02 (0.99–1.05)
	Neither	726/1162	65%	Reference
Attempt to Quit (Yes vs. No)	Both Asked and Advised	1177/2219	54%	1.76 (1.75–1.77)
	Either Asked or Advised	359/735	47%	1.60 (1.57–1.63)
	Neither	549/1179	46%	Reference
Pharmacological quit methods (Yes vs. No)	Both Asked and Advised	488/1177	40%	1.99 (1.97–2.02)
	Either Asked or Advised	109/359	31%	1.56 (1.49–1.63)
	Neither	111/549	18%	Reference
Non-pharmacological quit methods (Yes vs. No)	Both Asked and Advised	137/1177	10%	2.01 (1.94–2.08)
	Either Asked or Advised	32/359	11%	2.91 (2.74–3.08)
	Neither	24/549	4%	Reference

**Notes:** Instrumental variable (IV) analysis was implemented using the two-stage residual inclusion (2SRI) method. The number of doctor office visits in the past 12 months was used as the IV. The analysis took into account the complex NHIS survey design. All models adjusted for the 16 covariates listed in Table 1.

**Table 3:** The association between provider–patient discussion and quit behavior from a propensity score weighted model and a multivariable logistic model.

Outcome	Exposure Variable: Level of Discussion	Propensity Score Weighted Model ORs (95% CI) ^a^	Multivariable Logistic Model OR (95% CI) ^b^
Intent to Quit (Yes vs. No)	Both Asked and Advised	1.74 (1.31–2.30)	1.68 (1.32–2.14)
	Either Asked or Advised	1.04 (0.77–1.414)	0.97 (0.75–1.25)
	Neither	Reference	Reference
Attempt to Quit (Yes vs. No)	Both Asked and Advised	1.88 (1.49–2.38)	1.80 (1.47–2.21)
	Either Asked or Advised	1.24 (0.94–1.64)	1.19 (0.92–1.55)
	Neither	Reference	Reference
Pharmacological quit methods (Yes vs. No)	Both Asked and Advised	2.01 (1.44–2.82)	1.95 (1.41–2.70)
	Either Asked or Advised	1.72 (1.12–2.64)	1.54 (1.00–2.36)
	Neither	Reference	Reference
Non-pharmacological quit methods (Yes vs. No)	Both Asked and Advised	1.92 (1.01–3.66)	1.97 (0.97–3.99)
	Either Asked or Advised	2.57 (1.17–5.67)	2.40 (1.09–5.30)
	Neither	Reference	Reference

**Notes:**^a^ SURVEYLOGISTIC regression using the product of survey weight and inverse probability of treatment weight while controlling for the 17 covariates listed in Table 1. ^b^ SURVEYLOGISTIC regression using survey weights while controlling for the 16 covariates listed in Table 1.

## Data Availability

Data used for this study were publicly available from the NIHS website, https://www.cdc.gov/nchs/nhis/data-questionnaires-documentation.htm (accessed on January 2020).

## Supplementary Materials

The following are available online at https://www.mdpi.com/article/10.3390/ijerph18094593/s1, Figure S1: Flow chart of the study population selection; Table S1: Unweighted characteristics of study sample by the 3-level of provider-patient discussion, 2015 NHIS; Table S2: Falsification test of total number of office visits in the past 12 months as an Instrumental Variable; Table S3: Characteristics of study sample by the level of discussion (survey and PS weights).
