# Supplementary material for: The Influence of Increasing Levels of Provider-Patient Discussion on Quit Behavior: An Instrumental Variable Analysis of a National Survey
Source: Int J Environ Res Public Health. 2021 Apr 26;18(9):4593. doi: 10.3390/ijerph18094593 (PMC8123707; doi:10.3390/ijerph18094593)
Supplement: Supplementary file 1 [file ijerph-18-04593-s001.zip › ijerph-1113268-supplementary.pdf]

Appendix Figure S1. Flow chart of the study population selection.

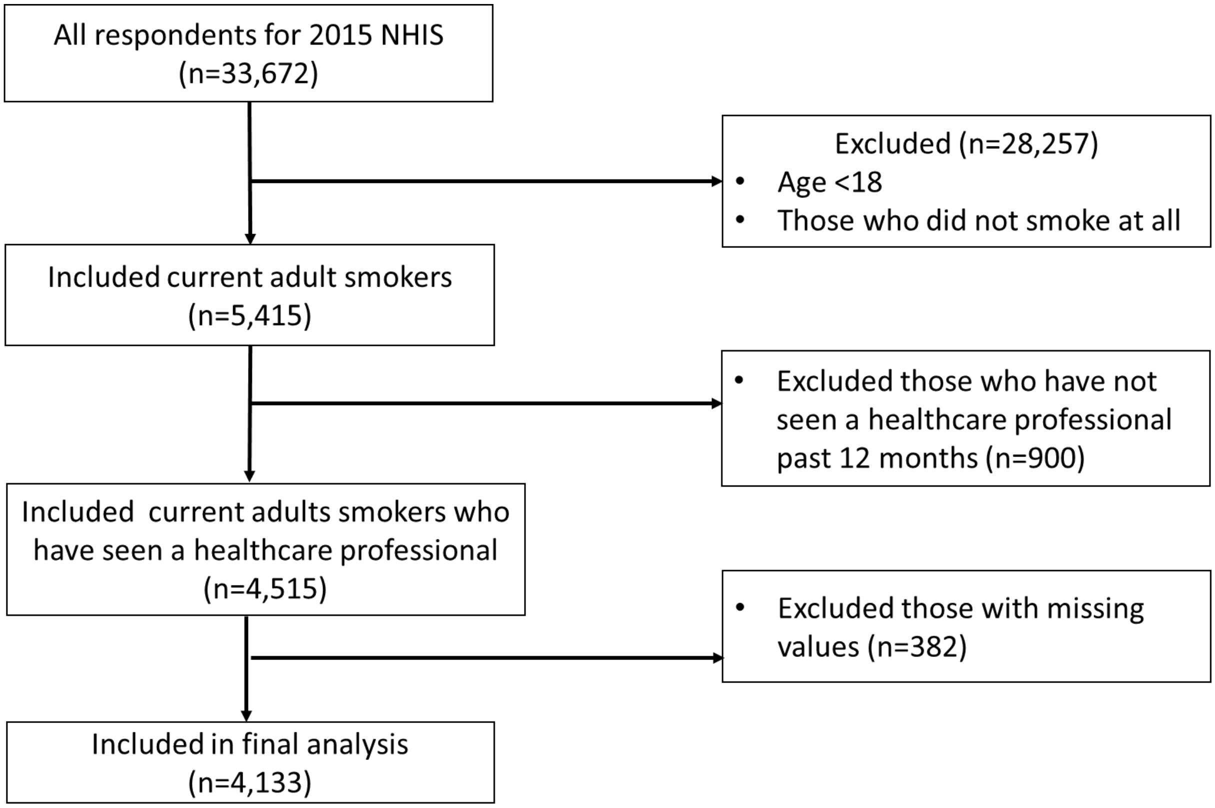

We first identified 5,415 adult (age≥18 years) current smokers in the 2015 NHIS data. Current smokers were defined as individuals who smoked at least 100 cigarettes in their lifetime and currently smoke cigarettes daily or on someday. We further restricted to those who had seen a doctor or other health professional in the past year, and excluding participants with missing values and those who responded "Don't know" or "Refused" for the main outcomes, exposure and covariates.

Appendix Table S1. Unweighted characteristics of study sample by the 3-level of provider-patient discussion, 2015 NHIS**.**

**Levels of discussion**	**Both Asked and Advised (N=2219)**	**Asked only or Advised only (N=735)**	**Neither Asked nor Advised (N=1179)**	
**Categorical Variables**	**N (unweighted%)**	**N (unweighted%)**	**N (unweighted%)**	**p-value**
**Sex**				0.0002
Female	1202 (54.17)	414 (56.33)	563 (47.75)	
Male	1017 (45.83)	321 (43.67)	616 (52.25)	
**Race/Ethnicity**				<.0001
White Non-Hispanic	1591 (71.70)	490 (66.67)	753 (63.87)	
Black Non-Hispanic	310 (13.97)	135 (18.37)	150 (12.72)	
Hispanic	197 (8.88)	68 (9.25)	172 (14.59)	
Others	121 (5.45)	42 (5.71)	104 (8.82)	
**Marital Status**[a]				0.0373
Yes	958 (43.17)	287 (39.05)	464 (39.36)	
No	1261 (56.83)	448 (60.95)	715 (60.64)	
**Region**				<.0001
Northeast	405 (18.25)	95 (12.93)	148 (12.55)	
Midwest	535 (24.11)	173 (23.54)	305 (25.87)	
South	772 (34.79)	287 (39.05)	408 (34.61)	
West	507 (22.85)	180 (24.49)	318 (26.97)	
**Education**				0.3846
Less than High School	538 (24.25)	205 (27.89)	284 (24.09)	
High School Graduate	598 (26.95)	191 (25.99)	300 (25.45)	
Some College	519 (23.39)	170 (23.13)	292 (24.77)	
College or Above	564 (25.42)	169 (22.99)	303 (25.70)	
**Employment**				<.0001
Yes	1083 (48.81)	397 (54.01)	737 (62.51)	
No	1136 (51.19)	338 (45.99)	442 (37.49)	
**Insurance**				<.0001
Private	806 (36.32)	288 (39.18)	505 (42.83)	
Medicaid	434 (19.56)	153 (20.82)	215 (18.24)	
Medicare	555 (25.01)	147 (20.00)	134 (11.37)	
Others	210 (9.46)	52 (7.07)	61 (5.17)	
Uninsured	214 (37.35)	95 (16.58)	264 (46.07)	
**Income/poverty ratio**				0.9789
<1.00	576 (25.96)	191 (25.99)	307 (26.04)	
1.00 - 1.99	569 (25.64)	194 (26.39)	288 (24.43)	
2.00 - 3.99	633 (28.53)	206 (28.03)	343 (29.09)	
4.00 and over	441 (19.87)	144 (19.59)	241 (20.44)	
**Lung Disease**[b]				<.0001
Yes	1279 (57.64)	312 (42.45)	386 (32.74)	
No	940 (42.36)	423 (57.55)	793 (67.26)	

**CVD**[c]				<.0001
Yes	310 (13.97)	65 (8.84)	63 (5.34)	
No	1909 (86.03)	670 (91.16)	1116 (94.66)	
**Cancer**[d]				<.0001
Tobacco Related	87 (3.92)	17 (2.31)	20 (1.70)	
Non-tobacco Related	152 (6.85)	43 (5.85)	33 (2.80)	
None	1980 (89.23)	675 (91.84)	1126 (95.50)	
**Serious psychological distress**[e]				<.0001
Yes (Kessler score≥13)	272 (12.26)	76 (10.34)	82 (6.96)	
No (Kessler score < 13)	1947 (97.74)	659 (89.66)	1097 (93.04)	
**Disability/limitation**[f]				<.0001
Yes	854 (38.49)	196 (26.67)	205 (17.39)	
No	1365 (61.51)	539 (73.33)	974 (82.61)	
**Continuous variables**	**Unweighted Mean (SD)**	**Unweighted Mean (SD)**	**Unweighted Mean (SD)**	**p-value**
**Age** (years)	50.1 (14.71)	46.09 (16.19)	42.7 (15.47)	<.0001
**Smoking length**[g] (year)	32.25 (15.37)	27.86 (16.32)	24.53 (15.74)	<.0001
**Number of cigarettes smoked daily**	13.16 (9.22)	11.1 (8.89)	9.91 (8.25)	<.0001

Notes:

a. Married included those who are married or living with a partner
b. Lung disease types included COPD, emphysema, and chronic bronchitis;
c. Cardiovascular diseases (CVD) included coronary heart disease, angina, stroke hypertension, heart attack, and other heart disease;
d. Tobacco-related cancer types included 12 tobacco-associated cancers as defined by the CDC: Lip, oral cavity, pharynx, Esophagus, Stomach, Colon and rectum, Liver, Pancreas, Larynx, Trachea, lung, bronchus, Cervix uteri, Kidney and renal pelvis, Urinary bladder, and Acute myeloid leukemia (Tong et al., 2006).
e. The Kessler Psychological Distress Scale consists of six questions that asks about feelings of sadness, nervousness, restlessness, worthlessness, hopelessness, and feeling like everything is an effort during the past 30 days. Participants were asked to respond on a Likert Scale ranging between 'None of the Time' (score = 0) to 'All of the time' (score = 4), and a cutoff of 13 was used to dichotomize the status of serious psychological distress.
f. Defined as having any functional limitations inclusive of all physical conditions.
g. Calculated as the difference between age at interview and age when smoking regularly.

Appendix Table S2. Falsification test of total number of office visits in the past 12 months as an Instrumental Variable

**Outcome**	**Asked and Advised**	**Asked or Advised**	**Neither**
Intent to Quit			
β	0.0734	-0.0258	0.0282
p-value	0.022*	0.596	0.510
sample size	2184	727	1162
Attempt to Quit			
β	0.0576	0.0809	-0.0127
p-value	0.055	0.047*	0.768
sample size	2219	735	1179

We performed first-stage F test and falsification test to check the validity of two assumptions for IV analysis. First, an IV must be correlated with the exposure variable; second, the IV must not be correlated with the outcome variable or any other unmeasured confounder such that the effect of the IV on the outcome is only through the exposure variable (Terza et al., 2008).

The first assumption is often tested using the first-stage F test, where an IV is not considered a weak instrument if the value of F-statistic is greater than 10.(Geraci et al., 2014; Terza et al., 2008) Since the second assumption cannot be directly tested through a confirmatory test, a falsification test is performed as an alternative to show empirically a lack of violation. Failure of falsification test (p-value<0.05) indicates that the second assumption may be implausible for the chosen IV (Keele et al., 2019).

The F test and falsification tests both showed that the number of doctor office visit is a reasonable instrument. F test showed that the number of office visit was strongly associated with the exposure (F test=87.61, P value <0.001). The falsification test was conducted in three groups for the two primary outcomes separately, using the approach proposed by Keel and colleagues (Keele et al., 2019). The results suggested that the IV passed the tests, except in the "both" group for the intent to quit (P= 0.022) and the "either" group for attempt to treat (p=0.047). However, because the magnitude of the estimate of IV (β) was very small, the IV had only a weak direct effect on the outcomes, thus would not affect the validity of the IV analysis in a consequential way.

Appendix Table S3. Characteristics of study sample by the level of discussion (survey and PS weights).

Levels of discussion	Asked and Advised (2A N=2219)	Asked or Advised (1A , N=735)	Neither Asked nor Advised (0A, N=1179)	
**Categorical Variables**	**N (weighted%)**	**N (weighted%)**	**N (weighted%)**	**p-value[a]**
**Sex**				0.919
Female	1202 (50.03)	414 (48.92)	563 (50.16)	
Male	1017 (49.97)	321 (51.08)	616 (49.84)	
**Race/Ethnicity**				0.965
White Non-Hispanic	1591 (73.66)	490 (73.13)	753 (75.2)	
Black Non-Hispanic	310 (12.76)	135 (13.11)	150 (11.57)	
Hispanic	197 (8.4)	68 (9.12)	172 (8.45)	
Others	121 (5.17)	42 (4.65)	104 (4.77)	
**Marital Status[b]**				0.975
Yes	958 (53.41)	287 (53.74)	464 (54.13)	
No	1261 (46.59)	448 (46.26)	715 (45.87)	
**Region**				0.625
Northeast	405 (16.8)	95 (15.7)	148 (20.75)	
Midwest	535 (27.93)	173 (28.75)	305 (25.85)	
South	772 (36.48)	287 (36.53)	408 (35.73)	
West	507 (18.79)	180 (19.01)	318 (17.67)	
**Education**				0.998
Less than High School	538 (25.28)	205 (24.81)	284 (24.86)	
High School Graduate	598 (26.92)	191 (28.09)	300 (26.37)	
Some College	519 (23.08)	170 (22.65)	292 (22.99)	
College or Above	564 (24.72)	169 (24.45)	303 (25.78)	
**Employment**				0.744
Yes	1083 (57.61)	397 (58.52)	737 (56.04)	
No	1136 (42.39)	338 (41.48)	442 (43.96)	
**Insurance**				0.996
Private	806 (44.98)	288 (45.58)	505 (43.01)	
Medicaid	434 (17.99)	153 (17.51)	215 (18.75)	
Medicare	555 (16.37)	147 (15.88)	134 (16.47)	
Others	210 (7.05)	52 (6.91)	61 (8.21)	
Uninsured	214 (13.61)	95 (14.12)	264 (13.56)	
**Income/poverty ratio**				0.987
<1.00	576 (20.62)	191 (20.93)	307 (22.93)	
1.00 - 1.99	569 (23.64)	194 (22.98)	288 (22.97)	
2.00 - 3.99	633 (30.72)	206 (31.08)	343 (30.24)	
4.00 and over	441 (25.02)	144 (25.01)	241 (23.86)	
**Lung Disease[c]**				0.914
Yes	1279 (44.17)	312 (43.05)	386 (44.39)	
No	940 (55.83)	423 (56.95)	793 (55.61)	
**CVD[d]**				0.596
Yes	310 (9.25)	65 (8.97)	63 (11.14)	

No	1909 (90.75)	670 (91.03)	1116 (88.86)	
**Cancer**[e]				0.993
Tobacco Related	87 (2.37)	17 (2.45)	20 (2.64)	
Non-tobacco Related	152 (4.9)	43 (4.76)	33 (4.4)	
None	1980 (92.72)	675 (92.8)	1126 (92.96)	
**Serious psychological distress**[f]				0.956
Yes (Kessler score≥13)	272 (11.03)	76 (11.06)	82 (10.49)	
No (Kessler score < 13)	1947 (88.97)	659 (88.94)	1097 (89.51)	
**Disability/limitation**[g]				0.625
Yes	854 (25.03)	196 (24.13)	205 (26.92)	
No	1365 (74.97)	539 (75.87)	974 (73.08)	
**Continuous variables**	**Weighted Mean (SD)**	**Weighted Mean (SD)**	**Weighted Mean (SD)**	**p-value**[a]
**Age** (years)	45.53 (14.93)	45.27 (15.57)	45.52 (15.83)	0.958
**Smoking length**[h] (year)	27.5 (15.32)	27.34 (15.93)	27.73 (16.3)	0.943
**Number of cigarettes smoked daily**	11.98 (8.75)	12.04 (10.89)	12.78 (10.63)	0.621

Notes:

a. The analysis took into account the NHIS survey design. Comparison of categorical variables by the 3-level provider-patient discussion was conducted using Rao-Scott Chi-square test in SURVEYFREQ procedure, and using F-test in SURVEYREG procedure.

b. Married included those who are married or living with a partner

c. Lung disease types included COPD, emphysema, and chronic bronchitis;

d. Cardiovascular diseases (CVD) included coronary heart disease, angina, stroke hypertension, heart attack, and other heart disease;

e. Tobacco-related cancer types included 12 tobacco-associated cancers as defined by the CDC: Lip, oral cavity, pharynx, Esophagus, Stomach, Colon and rectum, Liver, Pancreas, Larynx, Trachea, lung, bronchus, Cervix uteri, Kidney and renal pelvis, Urinary bladder, and Acute myeloid leukemia (Tong et al., 2006).

f. The Kessler Psychological Distress Scale consists of six questions that asks about feelings of sadness, nervousness, restlessness, worthlessness, hopelessness, and feeling like everything is an effort during the past 30 days. Participants were asked to respond on a Likert Scale ranging between 'None of the Time' (score = 0) to 'All of the time' (score = 4), and a cutoff of 13 was used to dichotomize the status of serious psychological distress.

g. Defined as having any functional limitations inclusive of all physical conditions.

h. Calculated as the difference between age at interview and age when smoking regularly.

References

Geraci, A., Fabbri, D., Monfardini, C., 2014. Testing Exogeneity of Multinomial Regressors in Count Data Models: Does Two Stage Residual Inclusion Work? SSRN Electronic Journal.

Keele, L., Zhao, Q., Kelz, R.R., Small, D., 2019. Falsification Tests for Instrumental Variable Designs With an Application to Tendency to Operate. Med Care 57:167-71.

Terza, J.V., Basu, A., Rathouz, P.J., 2008. Two-stage residual inclusion estimation: addressing endogeneity in health econometric modeling. J Health Econ 27:531-43.

Tong, E.K., Ong, M.K., Vittinghoff, E., Pérez-Stable, E.J., 2006. Nondaily Smokers Should Be Asked and Advised to Quit. American Journal of Preventive Medicine 30:23-30.
